# Nimesulide Improves the Symptomatic and Disease Modifying Effects of Leflunomide in Collagen Induced Arthritis

**DOI:** 10.1371/journal.pone.0111843

**Published:** 2014-11-06

**Authors:** Ahmed M. Al-Abd, Fahad A. Al-Abbasi, Salwa M. Nofal, Amani E. Khalifa, Richard O. Williams, Wafaa I. El-Eraky, Ayman A. Nagy, Ashraf B. Abdel-Naim

**Affiliations:** 1 Department of Pharmacology and Toxicology, Faculty of Pharmacy, King Abdulaziz University, Jeddah, Saudi Arabia; 2 Department of Biochemistry, Faculty of Science, King Abdulaziz University, Jeddah, Saudi Arabia; 3 Department of Pharmacology, Medical Division, National Research Centre, Giza, Egypt; 4 Department of Pharmacology and Toxicology, Faculty of Pharmacy, Ain Shams University, Cairo, Egypt; 5 Kennedy Institute of Rheumatology, University of Oxford, Oxford, United Kingdom; 6 Department of Forensic Medicine and Clinical Toxicology, Faculty of Medicine, Tanta University, Egypt; Veterans Affairs Medical Center, United States of America

## Abstract

Nimesulide is a COX-2 inhibitor used for symptomatic relief of rheumatoid arthritis. Leflunomide is an anti-pyrimidine used to manage the progression of rheumatoid arthritis. Herein we studied the influence of nimesulide and leflunomide combination in terms of disease symptoms and progression using collagen-induced arthritis model in mice, as a model for rheumatoid arthritis. Collagen induced arthritis was induced by immunization with type II collagen. Assessment of joint stiffness and articular hyperalgesia were evaluated using a locomotor activity cage and the Hargreaves method, respectively. Disease progression was assessed via arthritic index scoring, X-ray imaging, myeloperoxidase enzyme activity and histopathologic examination. Nimesulide induced only transient symptomatic alleviation on the top of decreased leucocytic infiltration compared to arthritis group. However, nimesulide alone failed to induce any significant improvement in the radiological or pathological disease progression. Leflunomide alone moderately alleviates the symptoms of arthritis and moderately retarded the radiological and pathological disease progression. Combination of nimesulide and leflunomide significantly improved symptomatic (analgesia and joint stiffness) and arthritic disease progression (radiological, pathological and Myeloperoxidase enzyme activity) in collagen induced arthritis animal model.

## Introduction

Rheumatoid arthritis (RA) is a chronic progressive systemic inflammatory disorder characterized by synovial inflammation, cartilage damage, progressive bone erosion, and articular functional disability. The world wide incidence of RA ranges from 0.5% to 1.0% and it is more prevalent in women compared to men [1]. Historically, non-steroidal anti-inflammatory drugs “NSAIDs” have been considered to be the primary treatment option for RA. Yet, NSAID failed to exert any significant delay in RA disease progression. Accordingly, disease modifying anti-rheumatic drugs “DMARDs” have become the first treatment option [2]. NSAIDs can mediate short term symptomatic amelioration, but with very poor long term outcome [3]. On the other hand, DMARD based regimens mainly aim to intervene in disease progression, with limited or no short term symptomatic alleviation. Several novel treatments have been tested or suggested for managing rheumatoid arthritis symptoms and/or disease progression, such as lymphocyte co-stimulation-targeted therapy [4], TNFα blocking agents [5], B-cell targeted therapy [6] and novel anti-inflammatory drugs with antioxidant activity [7]. However, the economic burden and patient compliance to injectable drugs limited the widespread use of these agents [8], [9].

Leflunomide (LEF) is a DMARD used for the treatment of several autoimmune disorders such as RA [10]. The active leflunomide metabolite, A771726LEF, is generated non-enzymatically or by hepatic microsomal enzymes (CYP 2C9) [11]. The active metabolite of leflunomide is considered to be dihydroorotate dehydrogenase (DHODH) enzyme inhibitor that decreases pyrimidine synthesis [12]. Yet, leflunomide is considered to be a selective anti- T cell agent for autoimmune disorders [13], [14]. Leflunomide possesses other advantageous anti-inflammatory effects, such as COX-2 inhibition, matrix metalloproteinase inhibition and anti-chemotaxis, [15]–[18].

Nimesulide (NIM) is a selective potent cycloxygenase-2 (COX-2) inhibitor [19]. Besides its COX-2 inhibitory activity, nimesulide inhibits several superoxide anion generating enzymes such as myeloperoxidase (MPO) [20]. Other anti-inflammatory properties for nimesulide have been reported such as, suppression of the expression of platelet activation factor (PAF), tumor necrosis factor-α and inhibition of matrix metalloproteinase enzymes [21]. In view of these properties, nimesulide is a strong candidate for combination therapy with DMARDs for the treatment for RA.

Previously, we found that nimesulide improved the disease ameliorating effect of methotrexate in the CIA model [22]. Herein, we extended our finding by studying the influence of nimesulide and leflunomide combination in terms of clinical severity and disease progression in CIA in mice.

## Results

### Symptomatic assessment of arthritis

**Figure 1:**
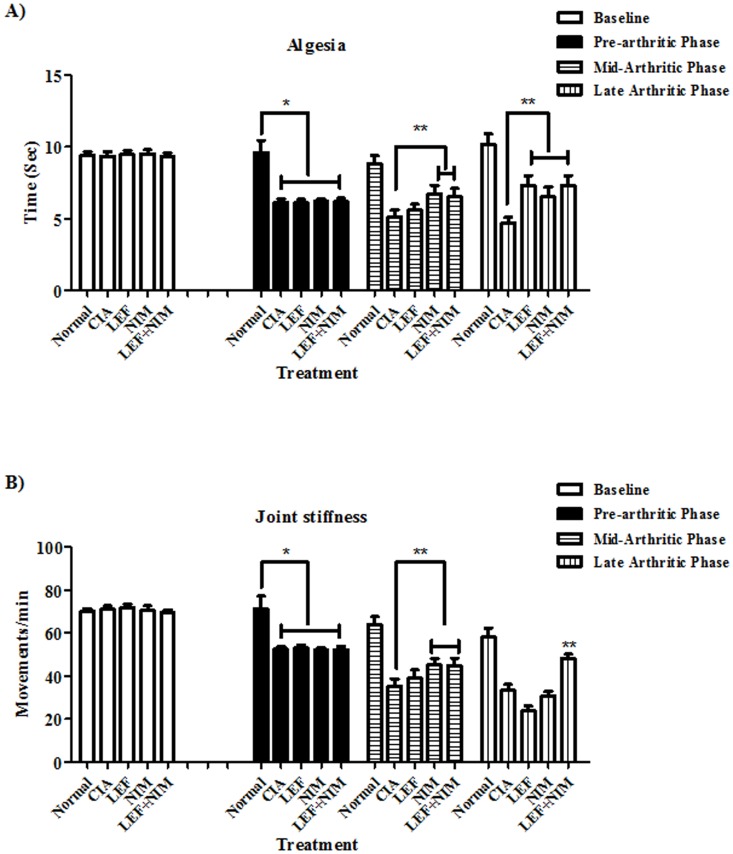
Symptomatic assessment of arthritis. Mice with CIA were treated with leflunomide (LEF), nimesulide (NIM), or leflunomide+nimesulide (LEF+NIM) and compared to untreated arthritic and normal mice. Hyperalgesia (A) and joint stiffness (B) were recorded at pre-arthritic, mid-arthritic and late arthritic phases of arthritis and compared to base line readings. Data are presented as mean ± SD. * Significantly different from normal mice; ** significantly different from CIA-mice.

The Hargreaves's method for assessing articular hyperalgesia was used herein to monitor joint algesia and to check for the potential effect of combining nimesulide to leflunomide in mice with CIA. Before treatment, the CIA control group manifested pre-arthritic shortening in withdrawal latency (WDL) and algesic response prior to the appearance of clinical signs of arthritis. At the mid-arthritic phase, nimesulide and nimesulide+leflunomide significantly prolonged the WDL compared to CIA control group. On contrary, at the late arthritic phase, all single (LEF or NIM) and combination treatment regimens induced significant analgesic effects in terms of prolonged WDL. LEF and LEF+NIM groups induced equal prolongation in WDL with 55.3% compared to the CIA-group. Treatment with nimesulide alone resulted in weaker analgesia manifested as WDL prolongation of only 38.3% relative to CIA-group. Interestingly, WDL of animals treated with leflunomide or leflunomide/nimesulide combination was non-significantly different from normal non arthritic mice at the late arthritic phase Fig. 1-A.

Joint stiffness is one of the main symptoms of RA, leading to a reduction in mobility. As a measure of this, we recorded the number of movements each animal made within the open field instrument. Locomotor activity of CIA mice started to decline in the pre-arthritic phase with further deterioration up to the late-arthritic phase. In the mid-arthritic phase, nimesulide transiently improved joint stiffness with a significant increase in the total number of movements per minute. Nimesulide treatment did not induce any further improvement in joint stiffness at the late-arthritic phase. Leflunomide alone did not induce any significant improvement in joint stiffness at the mid-arthritic or late-arthritic phases. leflunomide+nimesulide combined treatment showed significant improvement in joint stiffness, which started from the mid-arthritic phase till the late-arthritic phase Fig. 1-B.

### Assessment of disease progression

**Figure 2:**
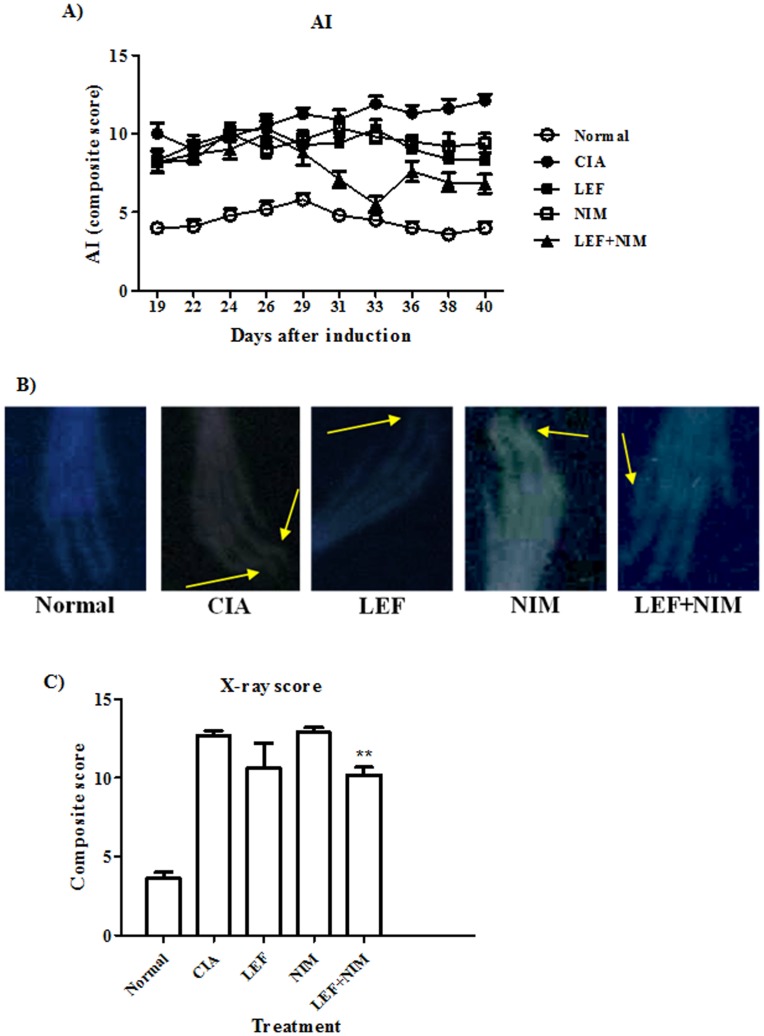
Assessment of disease progression. Mice with CIA were treated with leflunomide (LEF: ▪), nimesulide (NIM: □), or leflunomide+nimesulide (LEF+NIM: ▴) and compared to untreated arthritic (○) and normal mice (•). Arthritic index (AI) of mice was recorded over the duration of 40 days (A). Hind paws of mice were x-ray imaged at day 40 (B) and given a radiological score (C) as shown in the methods section. Data are presented as mean ± SD. ** Significantly different from CIA-mice.

Arthritic index (AI) is a semi quantitative parameter that reflecting the severity of polyarthritis. Animals showed stable, low grade, and persistent polyarthritis after immunization with collagen. Animals treated with leflunomide+nemisulide showed the fastest onset of improvement in AI (31 days after the primary induction of arthritis). Animals treated with leflunomide or nimesulide alone started to show improvement in AI at day 35 after induction. After 40 days of induction, the reduction in AI scores was found to be 31.4%, 22.3%, and 43.8% in LEF-group, NIM-group, and (LEF+NIM)-group respectively compared to the CIA controlgroup Fig. 2-A.

After 40 days of primary immunization (arthritis induction), articular damage was assessed using diagnostic X-ray imaging. Significant periostitis, bone erosion, joint malalignment and cartilaginous deterioration was observed in CIA group indicative of severe articular damage. These radiological changes were also assessed in all other treatment groups Fig. 2-B. Nimesulide treatment alone did not show any radiological improvement. On the other hand, treatment with leflunomide improved the total radiological score by 16.5%, while treatment with leflunomide and nimesulide together provided 19.1% radiological protection Fig. 2-C.

In terms of detailed radiological assessment, leflunomide treatment significantly improved bone erosion, cartilage depth and joint alignment by 21.1%, 23.5% and 17.2%, respectively, compared to the CIA control group. However, combined leflunomide+nimesulide treatment significantly improved all radiological parameters compared to the CIA group. In addition, leflunomide+nimesulide treatment significantly improved bone erosion and periostitis compared to the LEF-group **Table 1**.

**Table 1:** Detailed radiographic evaluation of articular damage.

Treatment group	CIA-group	LEF-group	NIM-group	LEF+NIM-group
**Bone erosion**	3.3±0.1	2.6^a^±0.3	3.6±0.2	2^a,b^±0.2
**Cartilage distance**	3.4±0.1	2.6^a^±0.5	3.1±0.2	2.9^a^±0.2
**Periostitis**	3.2±0.2	3±0.3	3.1±0.2	2.6^a,b^±0.2
**Joint alignment**	2.9±0.1	2.4^a^±0.3	3.1±0.2	2.9^a^±0.3

Data are presented as mean ± SEM.

n = 10.

a: significantly different from CIA-group at *p*<0.05.

b: significantly different from LEF-group at *p*<0.05.

### Histopathological assessment of articular damage

**Figure 3:**
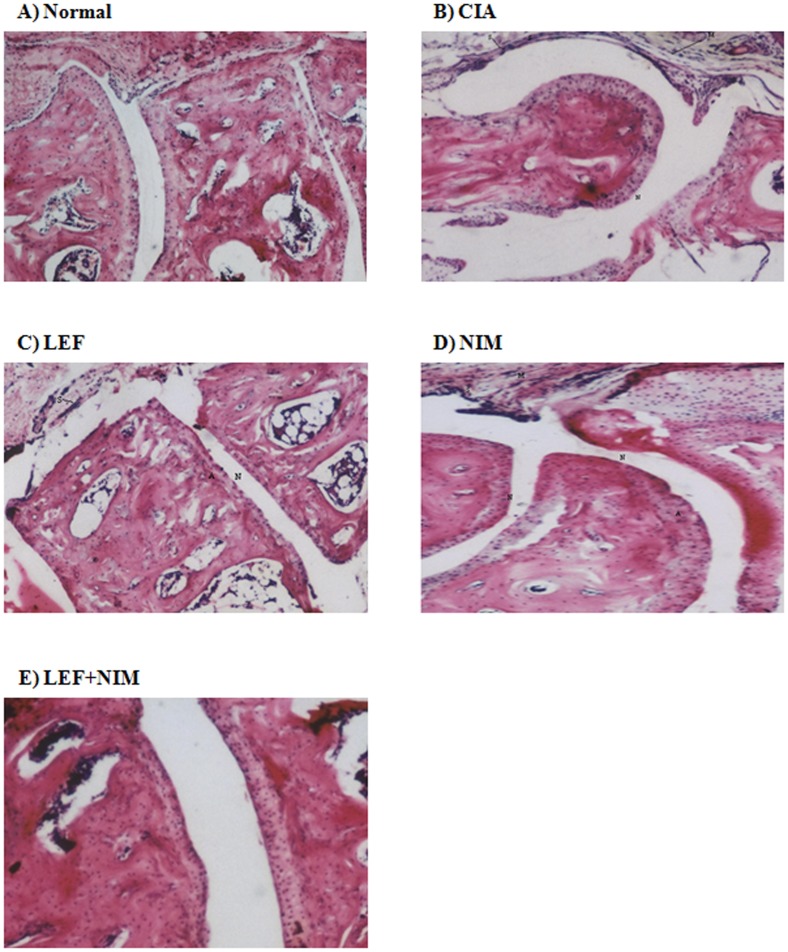
Histopathological assessment of articular damage. Mice with CIA were treated with leflunomide (LEF: Panel C), nimesulide (NIM: Panel D), or leflunomide+nimesulide (LEF+NIM: Panel E) for 40 days and the H&E stained joints were compared with untreated arthritic (CIA: Panel B) and normal mice (Panel A). Pathological findings were compared in terms of synovial hyperplasia (S), articular irregularity (A), narrowing of joint space (N), and lymphocytic infiltration (M).

Articular damage was further confirmed pathologically in H&E stained sagittal sections in the joints of mice. CIA resulted in severe hyperplasia in the synovial membrane, irregularity and roughness of the articular surface, and narrowing of joint space as well as excessive leucocytic infiltration Fig. 3-A&B. Treatment with nimesulide alone resulted in decreased intra-articular leucocytic infiltration Fig. 3-D. Synovial membrane hyperplasia, articular surface roughness and narrowing of the joint space were decreased by leflunomide treatment Fig. 3-C. Of all the treatment groups, leflunomide+nimesulide showed the greatest histopathological improvement compared to the CIA group Fig. 3-E.

**Figure 4:**
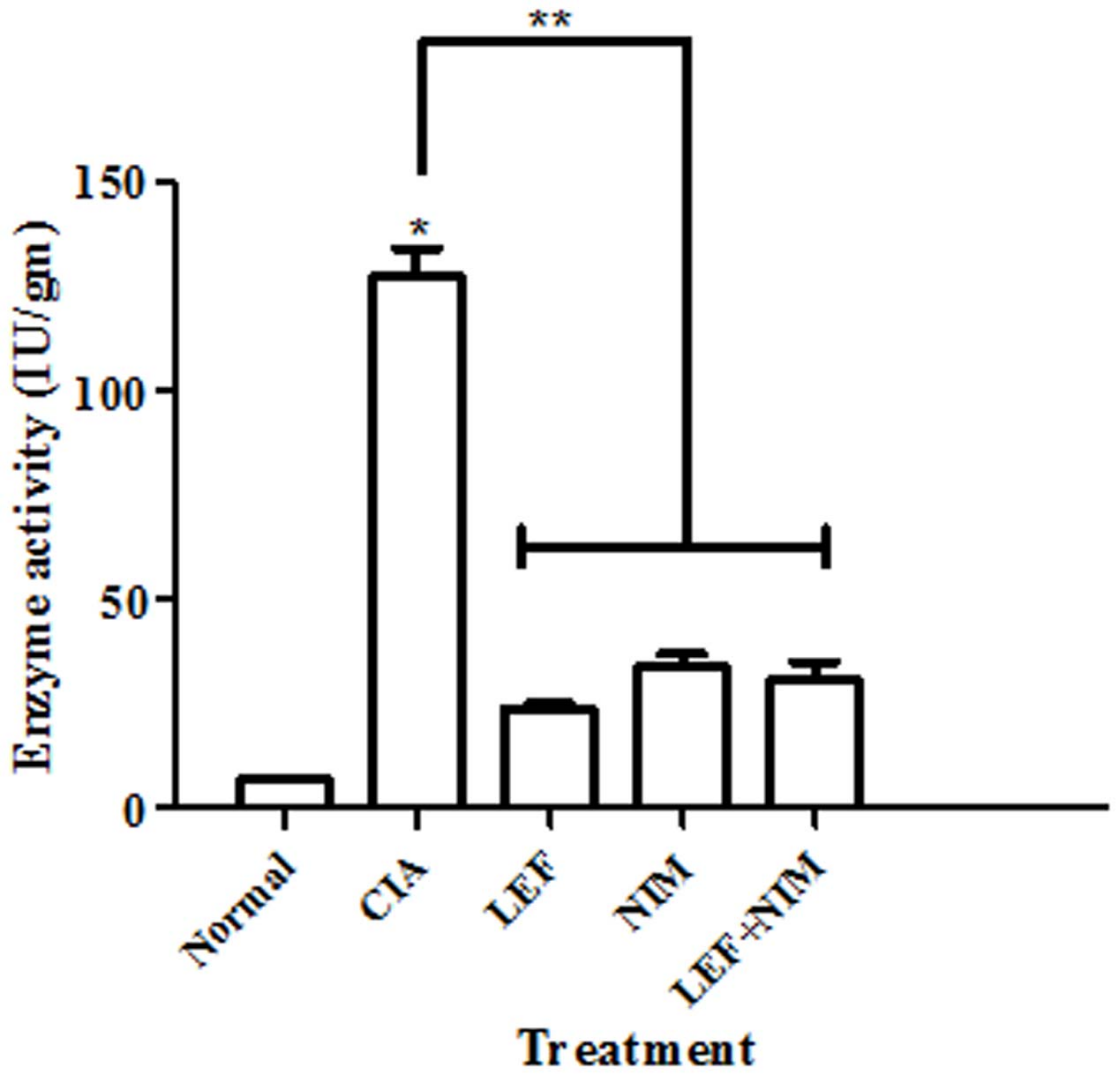
Intra-articular MPO activity. Mice with CIA were treated with leflunomide (LEF), nimesulide (NIM), or leflunomide+nimesulide (LEF+NIM) and compared to untreated arthritic and normal mice. Articular MPO activity was measured 40 days after treatment as a marker of leucocytic infiltration.

RA is characterized by leucocytic infiltration and the presence of neutrophils is often regarded as a marker of active disease. Herein, the activity of myeloperoxidase (MPO) was used as a marker for neutrophilic infiltration in the joints of mice with CIA. After 40 days of the primary induction of arthritis, the MPO level was elevated in the joints of mice with CIA-mice. However, all single and combination treatments significantly decreased the MPO activity compared to the CIA group (Fig. 4).

## Discussion

Treatment options for RA warrants a lot of visionary decisions; it is not only confined to symptomatic alleviation [23]. NSAID's provide partial symptomatic alleviation in RA; however they do not generally induce long term joint protection. Nimesulide shows strong anti-inflammatory activity beyond COX-2 inhibition [24], [25]. On the other hand DMARDS such as, leflunomide, can induce significant retardation in RA disease progression but they are relatively weak in improving key symptoms of RA such as joint stiffness and joint pain [3]. Hence, a DMARD/NSAID combination may provide long term prevention of disease progression on the top of short term symptomatic control [26]. In the current study, we have investigated the added value of combining nimesulide with leflunomide in controlling experimental arthritis.

Amelioration of the major symptoms of RA such as algesia and joint stiffness is necessary for patient compliance [27]. NSAID's are the major drug category used for this purpose [26]. Despite the huge number of existing NSAIDs, new agents with pharmacological properties and/or less toxic side effects are introduced to the field [7]. In our study, nimesulide alone alleviated joint pain at both the mid- and late arthritic phases (day 21 and day 40 after primary arthritis induction, respectively). However, nimesulide alone temporarily alleviated joint stiffness at mid-arthritic phase and failed at the late arthritic phase. Nimesulide is strong anti-inflammatory analgesic drug that possesses more than just COX-2 inhibition activity [24], [25]. That might explain its ability for pain alleviation at all arthritic stages [28]; in addition, the transient improvement in joint stiffness might be partly attributed to the analgesic effect [22]. Leflunomide alone failed to induce any significant improvement in joint stiffness. This might be explained by the suboptimal dose level of leflunomide (3.75 mg/kg weekly) used in the current study compared to 10 mg/kg daily in other studies [29]. However, leflunomide at the current dose exerted some pain alleviation effect at the late arthritic phase which might be directly attributed to its moderate anti-inflammatory effects or secondary to its robust disease modifying anti-rheumatic activity [30]. The combination of nimesulide and suboptimal dose of leflunomide could be considered tentative evidence of a synergistic interaction which significantly improved both algesic and joint stiffness signs of CIA at the mid- and late arthritic stages. Symptomatic enhancement effect could be permeated partly to the shared COX-2 inhibition activity of both agents [16], [19].

With respect to disease progression, leflunomide+nimesulide induced significant suppression of CIA progression in terms of time and magnitude. At day 33, the AI of LEF+NIM group was not significantly different from normal animals. In addition to the AI, the radiological evaluation showed significant protection for the leflunomide+nimesulide combination compared to either agent alone. Radiological progression is an important feature of RA, as well as CIA [31]. Despite the use of a sub-optimal dose of leflunomide in the current study, combination with nimesulide showed significant improvement in all aspects of the radiological evaluation of arthritis. Moreover, periostitis and bone erosion were significantly improved compared to the LEF-group. This strengthens the tentative synergistic interaction between leflunomide and nimesulide not only on the symptomatic level, but also on the disease modifying level [32].

The enhancement effect of nimesulide to leflunomide was further examined histopathologically. Nimesulide alone, significantly blunted the neutrophilic infiltration within joints of mice with CIA as shown by decreased MPO to almost normal levels. MPO is an enzyme known to be highly abundant to the azurophilic granules of neutrophils [33]. The role of neutrophil involvement in CIA and joint damage is well established. Yet, the anti-chemotaxis effect of nimesulide might partly explain the synergistic interaction with leflunomide in terms of disease progression [25], [34].

## Conclusions

Nimesulide enhances the symptomatic, clinical and radiological anti rheumatic activity of Leflunomide in mice with CIA.

## Materials and Methods

### Chemicals and Drugs

Collagen Type II was prepared as previously described from bovine cartilage [35]. Complete Freund Adjuvant (CFA), leflunomide, hexadecyl trimethylammonium bromide (HTAB), and *o*-dianisidin were purchased from Sigma-Aldrich Chemical Company (St. Louis, MO, USA). Nimesulide was gifted from Alkan Pharmaceutical Co., (6^th^ of October city, Egypt).

### Arthritis induction

Male Swiss albino mice (7 weeks old, 30 g weight) were bred in the animal house of the National Research Center (Dokki, Giza, Egypt). Mice were left for one week to acclimatize in the local animal house facility of the Department of Pharmacology prior to experimentation. Animals were kept at controlled environmental conditions with 12 h day/night cycles (20±4°C and 65±10% relative humidity) during the whole experiment. Standardized food pellets and water were supplied *ad libitum*.

Collagen type II solution (4 mg/ml) was prepared by dissolution in 0.1 M acetic acid. Collagen solution was emulsified with an equal volume of CFA. A Volume of 100 µl of final emulsion was injected intradermally (day 0). Only animals with positive signs of arthritis after 2 weeks of induction (87% of animals developed arthritis) were assigned randomly into 5 treatment groups (n = 10 animals/group). After 7 days of the primary induction, another dose (100 µg collagen in CFA) was injected intradermally [36]. The LEF-group received leflunomide 3.75 mg/kg i.p every week; NIM-group received nimesulide 20 mg/kg i.p. every two day; and LEF+NIM-group received leflunomide (3.75 mg/kg i.p) every week and nimesulide (20 mg/kg i.p.) daily. All treatments started 7 days after induction. Treatment groups were compared to mice with CIA (CIA-group) that received no treatment and to a normal non-immunised group (Normal-group).

The Animal Care and Use Committee (IACUC) of the National Research Center (Animal Rights and ethics Committee) approved this complete study.

### Assessment of arthritis severity

Arthritic index (AI) was recorded as previously described [37]. Briefly, mice paws were inspected every other day and AI was scored in each paw as follows: erythema and slight swelling in a single digit is given 0.5; erythema and slight swelling in two or more digits is given 1; erythend ma mild swelling in the whole limb is given 2; 3 =  erythema and gross swelling in the whole limb is given 3; limb disability and massive limb deformity is given 4. Composite AI equals the sum of each animal's four paws. AI scores were assessed blindly by 2 independent observers starting 2 days before the primary induction of arthritis until the end of the experiment.

### Assessment of articular hyperalgesia

Articular hyperalgesia was measured by Hargreaves method with minor in-house modifications [38]; briefly, mice were let to adapt in a designated chamber of planter test (Ugo Basile, Comerio, Italy) for 30 min. Inflamed joints were irradiated with an IR-beam from a movable bulb (Ugo Basile, Comerio, Italy) to induce algesia. The time taken for each animal to lick/withdraw IR-challenged limb was recorded and designated as withdrawal latency (WDL) [39]. The base-line for articular hyperalgesia was determined (2 days before the primary induction of arthritis) and compared to pre-arthritic phase (10 days after the primary induction of arthritis); mid-arthritic phase (25 days after the primary induction of arthritis); and late-arthritic phase (40 days after the primary induction of arthritis).

### Assessment of joint stiffness

Joint stiffness was correlated to the locomotor activity of animlas and measured using open field activity cage method. As previously described [39], animals were left for five minutes in the grid floor activity cage (Ugo Basile, Comerio, Italy). The animals' movements with their paws were converted into electric signals and the number of movements per minute was recorded. The base-line for locomotor activity was recorded (2 days before the primary induction of arthritis) and compared to pre-arthritic phase (10 days after the primary induction of arthritis); mid-arthritic phase (25 days after induction); and late-arthritic phase (40 days after the primary induction of arthritis).

### Determination of myeloperoxidase enzyme activity

MPO activity was determined in paws at the late-arthritic phase (40 days after the primary induction of arthritis) as previously described [40]. Briefly, mice were sacrificed; paws were immediately excised, weighed and rapidly homogenized for 15 minutes (0.5% hexadecyl trimethylammonuim bromide (HTAB) in 50 mM phosphate buffer, pH 6). Articular tissue homogenate (10% w/v) was centrifuged and 2.9 ml *o*-dianisidin (0.167 mg/ml) was added to 100 µL of the clear supernatant. Hydrogen peroxide (0.0005%) was finally added and absorbance was measured every 15 sec for 2 min (λ_max_ 460 nm). The rate of absorbance change was used to calculate the MPO activity per gram tissue (Molar absorbtivity of the color adduct is 1.13×10^4^). [33].

### X-rays analysis

Radiological assessment for joint damage was undertaken by X-ray imaging at the late-arthritic phase. Briefly, mice were sacrificed; anterior and hind limbs were excised and fixed in 10% buffered formalin solution. Paws were placed on an X-ray cassette (Medivance Instrument Limited, London, UK) and X-ray images were taken at 40 KV, 0.04 mA sec (Pioneer road-S-240 Salt Lake city, UT, USA). Radiological assessment for joint damage was performed as previously described [41]. Briefly, each limb's X-ray film was scored from 0–4 (4 is the worst) with respect to joint alignment, cartilage distance, bone erosion, and periostitis. The composite scores of each X-ray image represent the total radiological score. Each film was assessed blindly by 3 different observers [42].

### Histopathological examination

Histological assessment for articular tissues was performed as follows. Paraformaldhyde fixed tissues were decalcified by EDTA and embedded in paraffin wax. Cross sagittal sections (5 µm) were obtained and after dewaxing and rehydration, sections were stained with H&E.

### Statistical analysis

Data are presented as average ± SEM. Significance was tested using ANOVA) with LSD post hoc test was used for testing the significance of parametric data. Significance of non-parametric data was determined using the Mann-Whitney U test. All statistical calculations were carried out by SPSS software for windows, version 17.0.0.; P<0.05 was taken as the cut off value for significance.

## References

[1] Helmick CG, Felson DT, Lawrence RC, Gabriel S, Hirsch R, et al (2008) Estimates of the prevalence of arthritis and other rheumatic conditions in the United States. Part I. Arthritis Rheum 58: 15–25. 18163481 10.1002/art.23177

[2] Weinblatt ME (1996) Rheumatoid arthritis: treat now, not later! Ann Intern Med 124: 773–774. 10.7326/0003-4819-124-8-199604150-00012 8633840

[3] Pincus T, O′Dell JR, Kremer JM (1999) Combination therapy with multiple disease-modifying antirheumatic drugs in rheumatoid arthritis: a preventive strategy. Ann Intern Med 131: 768–774. 10577301 10.7326/0003-4819-131-10-199911160-00009

[4] Falgarone G, Semerano L, Rulle S, Boissier MC (2009) Targeting lymphocyte activation to treat rheumatoid arthritis. Joint Bone Spine 76: 327–332. 19535279 10.1016/j.jbspin.2008.12.007

[5] Wiens A, Correr CJ, Pontarolo R, Venson R, Quinalha JV, et al (2009) A systematic review and meta-analysis of the efficacy and safety of etanercept for treating rheumatoid arthritis. Scand J Immunol 70: 337–344. 19751268 10.1111/j.1365-3083.2009.02296.x

[6] Bracewell C, Isaacs JD, Emery P, Ng WF (2009) Atacicept, a novel B cell-targeting biological therapy for the treatment of rheumatoid arthritis. Expert Opin Biol Ther 9: 909–919. 19522556 10.1517/14712590903033919

[7] Khalil NA, Ahmed EM, El-Nassan HB, Ahmed OK, Al-Abd AM (2012) Synthesis and biological evaluation of novel pyrazoline derivatives as anti-inflammatory and antioxidant agents. Arch Pharm Res 35: 995–1002. 22870808 10.1007/s12272-012-0606-9

[8] Schipper LG, Kievit W, den Broeder AA, van der Laar MA, Adang EM, et al (2011) Treatment strategies aiming at remission in early rheumatoid arthritis patients: starting with methotrexate monotherapy is cost-effective. Rheumatology (Oxford) 50: 1320–1330. 21371999 10.1093/rheumatology/ker084

[9] Benucci M, Li Gobbi F, Sabadini L, Saviola G, Baiardi P, et al (2009) The economic burden of biological therapy in rheumatoid arthritis in clinical practice: cost-effectiveness analysis of sub-cutaneous anti-TNFalpha treatment in Italian patients. Int J Immunopathol Pharmacol 22: 1147–1152. 20074482 10.1177/039463200902200434

[10] Bartlett RR, Brendel S, Zielinski T, Schorlemmer HU (1996) Leflunomide, an immunorestoring drug for the therapy of autoimmune disorders, especially rheumatoid arthritis. Transplant Proc 28: 3074–3078. 8962190

[11] Rozman B (2002) Clinical pharmacokinetics of leflunomide. Clin Pharmacokinet 41: 421–430. 12074690 10.2165/00003088-200241060-00003

[12] Fox RI (1998) Mechanism of action of leflunomide in rheumatoid arthritis. J Rheumatol Suppl 53 20–26. 9666414

[13] Dimitrova P, Skapenko A, Herrmann ML, Schleyerbach R, Kalden JR, et al (2002) Restriction of de novo pyrimidine biosynthesis inhibits Th1 cell activation and promotes Th2 cell differentiation. J Immunol 169: 3392–3399. 12218161 10.4049/jimmunol.169.6.3392

[14] Fox RI, Herrmann ML, Frangou CG, Wahl GM, Morris RE, et al (1999) Mechanism of action for leflunomide in rheumatoid arthritis. Clin Immunol 93: 198–208. 10600330 10.1006/clim.1999.4777

[15] Kent EF Jr, Crawford J, Cohen HJ, Buckley RH (1990) Development of multiple monoclonal serum immunoglobulins (multiclonal gammopathy) following both HLA-identical unfractionated and T cell-depleted haploidentical bone marrow transplantation in severe combined immunodeficiency. J Clin Immunol 10: 106–114. 2338452 10.1007/BF00918192

[16] Hamilton LC, Vojnovic I, Warner TD (1999) A771726, the active metabolite of leflunomide, directly inhibits the activity of cyclo-oxygenase-2 in vitro and in vivo in a substrate-sensitive manner. Br J Pharmacol 127: 1589–1596. 10455314 10.1038/sj.bjp.0702708 PMC1566153

[17] Burger D, Begue-Pastor N, Benavent S, Gruaz L, Kaufmann MT, et al (2003) The active metabolite of leflunomide, A77 1726, inhibits the production of prostaglandin E(2), matrix metalloproteinase 1 and interleukin 6 in human fibroblast-like synoviocytes. Rheumatology (Oxford) 42: 89–96. 12509619 10.1093/rheumatology/keg038

[18] Cao WW, Kao PN, Aoki Y, Xu JC, Shorthouse RA, et al (1996) A novel mechanism of action of the immunomodulatory drug, leflunomide: augmentation of the immunosuppressive cytokine, TGF-beta 1, and suppression of the immunostimulatory cytokine, IL-2. Transplant Proc 28: 3079–3080. 8962191

[19] Sengupta S (1998) Cyclooxygenase-2: a new therapeutic target. Indian J pharmacology 31: 322–332.

[20] Bevilacqua M, Vago T, Baldi G, Renesto E, Dallegri F, et al (1994) Nimesulide decreases superoxide production by inhibiting phosphodiesterase type IV. Eur J Pharmacol 268: 415–423. 7805766 10.1016/0922-4106(94)90067-1

[21] Pelletier JP, Martel-Pelletier J (1993) Effects of nimesulide and naproxen on the degradation and metalloprotease synthesis of human osteoarthritic cartilage. Drugs 46 Suppl 1 34–39. 7506192 10.2165/00003495-199300461-00008

[22] Al-Abd AM, Inglis JJ, Nofal SM, Khalifa AE, Williams RO, et al (2010) Nimesulide improves the disease modifying anti-rheumatic profile of methotrexate in mice with collagen-induced arthritis. Eur J Pharmacol 644: 245–250. 20643120 10.1016/j.ejphar.2010.07.006

[23] Walker-Bone K, Farrow S (2007) Rheumatoid arthritis. Clin Evid (Online) 2007 PMC2943775 19454108

[24] Capecchi PL, Ceccatelli L, Beermann U, Laghi Pasini F, Di Perri T (1993) Inhibition of neutrophil function in vitro by nimesulide. Preliminary evidence of an adenosine-mediated mechanism. Arzneimittelforschung 43: 992–996. 8240466

[25] Tool AT, Mul FP, Knol EF, Verhoeven AJ, Roos D (1996) The effect of salmeterol and nimesulide on chemotaxis and synthesis of PAF and LTC4 by human eosinophils. Eur Respir J Suppl 22 141s–145s. 8871060

[26] Ramiro S, Radner H, van der Heijde D, van Tubergen A, Buchbinder R, et al (2011) Combination therapy for pain management in inflammatory arthritis (rheumatoid arthritis, ankylosing spondylitis, psoriatic arthritis, other spondyloarthritis). Cochrane Database Syst Rev CD008886. 21975788 10.1002/14651858.CD008886.pub2 PMC12416524

[27] Inotai A, Meszaros A (2012) Determinants of NSAID choice in rheumatoid arthritis–a drug utilization study. Acta Pol Pharm 69: 773–777. 22876621

[28] da Costa Araujo FA, de Santana Santos T, de Morais HH, Laureano Filho JR, de Oliveira ESED, et al (2012) Comparative analysis of preemptive analgesic effect of tramadol chlorhydrate and nimesulide following third molar surgery. J Craniomaxillofac Surg 40: e346–349. 22421470 10.1016/j.jcms.2012.01.018

[29] Coulthard LG, Costello J, Robinson B, Shiels IA, Taylor SM, et al (2011) Comparative efficacy of a secretory phospholipase A2 inhibitor with conventional anti-inflammatory agents in a rat model of antigen-induced arthritis. Arthritis Res Ther 13: R42. 21401925 10.1186/ar3278 PMC3132024

[30] Curnock AP, Robson PA, Yea CM, Moss D, Gadher S, et al (1997) Potencies of leflunomide and HR325 as inhibitors of prostaglandin endoperoxide H synthase-1 and -2: comparison with nonsteroidal anti-inflammatory drugs. J Pharmacol Exp Ther 282: 339–347. 9223572

[31] Joosten LA, Helsen MM, Saxne T, van De Loo FA, Heinegard D, et al (1999) IL-1 alpha beta blockade prevents cartilage and bone destruction in murine type II collagen-induced arthritis, whereas TNF-alpha blockade only ameliorates joint inflammation. J Immunol 163: 5049–5055. 10528210

[32] Williams RO (1998) Combination therapy in mice: what can we learn that may be useful for understanding rheumatoid arthritis? Springer Semin Immunopathol 20: 165–180. 9836375 10.1007/BF00832005

[33] Bradley PP, Priebat DA, Christensen RD, Rothstein G (1982) Measurement of cutaneous inflammation: estimation of neutrophil content with an enzyme marker. J Invest Dermatol 78: 206–209. 6276474 10.1111/1523-1747.ep12506462

[34] de Mello SB, Laurindo IM, Cossermelli W (1994) Action of the 4-nitro-2-phenoximethanesulphonanilide (nimesulide) on neutrophil chemotaxis and superoxide production. Sao Paulo Med J 112: 489–494. 7871312 10.1590/s1516-31801994000100003

[35] Miller EJ, Rhodes RK (1982) Preparation and characterization of the different types of collagen. Methods Enzymol 82 Pt A: 33–64. 10.1016/0076-6879(82)82059-4 7078441

[36] Cook AD, Braine EL, Campbell IK, Rich MJ, Hamilton JA (2001) Blockade of collagen-induced arthritis post-onset by antibody to granulocyte-macrophage colony-stimulating factor (GM-CSF): requirement for GM-CSF in the effector phase of disease. Arthritis Res 3: 293–298. 11549370 10.1186/ar318 PMC64841

[37] Urakawa K, Mihara M, Suzuki T, Kawamura A, Akamatsu K, et al (2000) Polyglutamation of antifolates is not required for induction of extracellular release of adenosine or expression of their anti-inflammatory effects. Immunopharmacology 48: 137–144. 10936511 10.1016/s0162-3109(00)00197-1

[38] Hargreaves K, Dubner R, Brown F, Flores C, Joris J (1988) A new and sensitive method for measuring thermal nociception in cutaneous hyperalgesia. Pain 32: 77–88. 3340425 10.1016/0304-3959(88)90026-7

[39] Chillingworth NL, Donaldson LF (2003) Characterisation of a Freund's complete adjuvant-induced model of chronic arthritis in mice. J Neurosci Methods 128: 45–52. 12948547 10.1016/s0165-0270(03)00147-x

[40] McVey DC, Vigna SR (2001) The capsaicin VR1 receptor mediates substance P release in toxin A-induced enteritis in rats. Peptides 22: 1439–1446. 11514026 10.1016/s0196-9781(01)00463-6

[41] Clark RL, Cuttino JT Jr, Anderle SK, Cromartie WJ, Schwab JH (1979) Radiologic analysis of arthritis in rats after systemic injection of streptococcal cell walls. Arthritis Rheum 22: 25–35. 365186 10.1002/art.1780220105

[42] Asanuma Y, Nagai K, Kato M, Sugiura H, Kawai S (2002) Weekly pulse therapy of methotrexate improves survival compared with its daily administration in MRL/lpr mice. Eur J Pharmacol 435: 253–258. 11821034 10.1016/s0014-2999(01)01555-2

